# The Reproducibility of Deep Learning-Based Segmentation of the Prostate Gland and Zones on T2-Weighted MR Images

**DOI:** 10.3390/diagnostics11091690

**Published:** 2021-09-16

**Authors:** Mohammed R. S. Sunoqrot, Kirsten M. Selnæs, Elise Sandsmark, Sverre Langørgen, Helena Bertilsson, Tone F. Bathen, Mattijs Elschot

**Affiliations:** 1 Department of Circulation and Medical Imaging, NTNU—Norwegian University of Science and Technology, 7030 Trondheim, Norway; kirsten.margrete.selnes@stolav.no (K.M.S.); tone.f.bathen@ntnu.no (T.F.B.); mattijs.elschot@ntnu.no (M.E.); 2 Department of Radiology and Nuclear Medicine, St. Olavs Hospital, Trondheim University Hospital, 7030 Trondheim, Norway; elise.sandsmark@stolav.no (E.S.); sverre.langorgen@stolav.no (S.L.); 3 Department of Cancer Research and Molecular Medicine, NTNU—Norwegian University of Science and Technology, 7030 Trondheim, Norway; helena.bertilsson@ntnu.no; 4 Department of Urology, St. Olavs Hospital, Trondheim University Hospital, 7030 Trondheim, Norway

**Keywords:** prostate, segmentation, deep learning, MRI, computer-aided diagnosis

## Abstract

Volume of interest segmentation is an essential step in computer-aided detection and diagnosis (CAD) systems. Deep learning (DL)-based methods provide good performance for prostate segmentation, but little is known about the reproducibility of these methods. In this work, an in-house collected dataset from 244 patients was used to investigate the intra-patient reproducibility of 14 shape features for DL-based segmentation methods of the whole prostate gland (WP), peripheral zone (PZ), and the remaining prostate zones (non-PZ) on T2-weighted (T2W) magnetic resonance (MR) images compared to manual segmentations. The DL-based segmentation was performed using three different convolutional neural networks (CNNs): V-Net, nnU-Net-2D, and nnU-Net-3D. The two-way random, single score intra-class correlation coefficient (ICC) was used to measure the inter-scan reproducibility of each feature for each CNN and the manual segmentation. We found that the reproducibility of the investigated methods is comparable to manual for all CNNs (14/14 features), except for V-Net in PZ (7/14 features). The ICC score for segmentation volume was found to be 0.888, 0.607, 0.819, and 0.903 in PZ; 0.988, 0.967, 0.986, and 0.983 in non-PZ; 0.982, 0.975, 0.973, and 0.984 in WP for manual, V-Net, nnU-Net-2D, and nnU-Net-3D, respectively. The results of this work show the feasibility of embedding DL-based segmentation in CAD systems, based on multiple T2W MR scans of the prostate, which is an important step towards the clinical implementation.

## 1. Introduction

Prostate cancer is the most detected cancer in men and the second most common cause of cancer related death for men worldwide [1]. An early diagnosis of prostate cancer is essential for a better disease management [2]. Following reasonable suspicion of prostate cancer, based on elevated prostate-specific antigen (PSA) levels in blood and a digital rectal examination (DRE), the patient, in many countries, is likely to be referred to a pre-biopsy magnetic resonance imaging (MRI) to guide the collection of biopsies [3]. To improve the diagnostic process, the use of multi-parametric MRI (mpMRI) has been established through international guidelines [4,5,6]. Additionally, mpMRI has been employed in active surveillance programs to follow up the patients with indolent lesions [7], prostate cancer risk calculators [8], and treatment response monitoring [6,9]. Currently, the mpMR images are interpreted qualitatively by a radiologist, which is a tedious, time-consuming [10], and reader opinion-dependent [11,12] process. The resulting vulnerability to inter and intra-observer variability is problematic for clinical applications based on multiple scans in time, such as with active surveillance and response monitoring, where reproducibility of results is paramount. Automated computer-aided detection and diagnosis (CAD) systems have the potential to overcome the limitations of the traditional radiological reading by implementing quantitative models to automate, standardize, and support reproducible interpretations of radiological images [10,13,14,15].

Segmentation is an essential step for prostate CAD systems [13,15]. It helps locate the volume of interest (VOI), enabling subsequent extraction of quantitative features for radiomics-based approaches. Accurate segmentation is paramount as the following steps of a CAD system are dependent on it. Traditionally, the VOI segmentation is performed manually by a radiologist on T2-weighted (T2W) MR images. However, deep learning (DL)-based segmentation methods have shown promising performances [16,17,18,19,20]. Importantly, the inter-observer variability between DL-based segmentation methods and expert radiologists has been shown to be approximately equal to that between expert radiologists [21]. However, little is known about the reproducibility of DL-based segmentation methods for clinical MRI scans. To investigate the reproducibility of DL-based segmentation, radiomics shape features can be used. Shape features, like prostate volume, are already part of today’s clinical risk calculators for prostate cancer [8] and will likely play an important role in future radiomics-based clinical applications.

Recently, the reproducibility of several radiomics features, using manually segmented masks of the prostate on clinical MR images, has been investigated [22,23,24,25]. The study by Schwier et al. [22] was the only one that used segmentations of the whole prostate gland and zones. Their results showed high reproducibility of shape features, between manual segmentations on mpMRI scans, during a short time interval. Despite the useful information provided by these studies, they do not provide insight into the reproducibility of automatically generated segmentations. Furthermore, these studies did not focus on investigating the reproducibility of the segmentation masks themselves, as the manual segmentations were considered the reference. To the best of our knowledge, the reproducibility of DL-based segmentations of the prostate, on clinical MR images, has not been previously investigated.

The contribution of this study is the assessment of the reproducibility of DL-based segmentations of the whole prostate gland (WP), peripheral zone (PZ), and the remaining prostate zones (non-PZ; central, transition, and anterior fibro-muscular zones, combined) by comparing radiomics shape features from T2W MR images acquired with short time intervals.

## 2. Materials and Methods

### 2.1. Dataset

In this study, we used an in-house collected mpMRI dataset from 244 patients (median age = 65; range: 44–76 years) for retrospective analysis. This dataset came from a previous prospective study conducted by our group. The patients were examined at St. Olavs Hospital, Trondheim University Hospital, Trondheim, Norway between March 2015 and December 2017, due to suspicion of prostate cancer, via the Norwegian standardized care pathway, in which patients with elevated PSA and/or abnormal DRE results are referred for an initial mpMRI scan to identify suspicious cancerous tissue [26]. If the radiologist detected suspicious tissue in the aforementioned prospective study, patients were randomly selected for either standard transrectal ultrasound-guided biopsy or in-bore targeted MR-guided biopsy. The latter group (*n* = 62), therefore, had two mpMRI scans. The Regional Committee for Medical and Health Research Ethics (REC Central Norway) approved the use of the dataset (identifiers 2013/1869 and 2017/576). All the patients signed informed consent prior to the initiation of the prospective study.

The dataset (T2W images) was split into a training set (*n* = 182), to train the DL-based segmentation networks, and an investigation set (*n* = 62), to investigate the reproducibility of shape features extracted from the segmented prostate masks. The investigation set was acquired at two different time points: first, at the initial visit for the detection of prostate cancer (scan 1) and second, during an MR-guided biopsy procedure (scan 2). The interval between scans ranged from 1–71 (median = 7) days. Patients in the collected dataset with two scans were assigned to the investigation set. Those who had only one scan, acquired at the initial visit for detection of prostate cancer, were assigned to the training set.

T2W MRI was performed on a Magnetom Skyra 3 T MRI system (Siemens Healthineers, Erlangen, Germany) with a turbo spin-echo sequence. The scanning parameters details are given in Table 1.

### 2.2. Prostate Segmentation

Manual segmentation of PZ and non-PZ for the in-house collected dataset was performed using ITK-SNAP [27] (version 3.6.0) by a radiology resident (E.S.) at St. Olavs Hospital, Trondheim University Hospital, Trondheim, Norway, under the supervision of a radiologist (S.L.) with more than 10 years’ experience in prostate imaging. PZ and non-PZ masks were used to generate the WP masks by merging. Lesion segmentation was beyond the scope of this study and was, therefore, not considered.

The DL-based segmentation was performed using three different convolutional neural networks (CNNs), which are all variants of the famous U-Net with an encoder-decoder scheme, along with long skip connections [28], further referred to as V-Net [18], nnU-Net-2D [16], and nnU-Net-3D [16]. These three networks were chosen for their popularity, good performance, and public availability. Unlike U-Net, V-Net is a residual learning network that replaces the maximum pooling operation with strided convolutions and the ReLU activation functions with PReLU activation functions [18]. On the other hand, nnU-Net does not introduce a new architecture, but it uses a 2D or 3D U-Net network with automatic self-configuration of pre-processing, network architecture, training, and post-processing [16]. nnU-Net-2D performed the segmentation on a 2D slice-by-slice basis, whereas V-Net and nnU-Net-3D performed the segmentation on a 3D volume basis. Prior to segmentation, all images were pre-processed in accordance with the corresponding segmentation method. The segmentation pre-processing, training, and testing were performed on a single NVIDIA Tesla P100 PCIe 16 GB GPU in Ubuntu 18.04.4 LTS.

V-Net was implemented with PyTorch [29] (version 1.4.0) using Python (version 3.6.9; Python Software Foundation, Wilmington, DE, USA) to generate two separate models for WP and PZ, which were used to generate non-PZ masks by subtraction. Each of the V-Net models was trained for 16,000 iterations with a batch size of 2. Adaptive moment estimation with momentum of 0.99, a weight decay of 1 × 10^−8^, and an initial learning rate of 1 × 10^-4^ were used for learning the network weights. The training time for each of the models was 10 h with cuDNN acceleration.

nnU-Net-2D (version 2.1) and nnU-Net-3D (version 2.1) were implemented with PyTorch (version 1.7.0) using Python (version 3.6.10) to generate one model for both PZ and non-PZ, which were used to generate the WP masks by merging. The nnU-Net-2D model was trained for 978 epochs with a batch size of 22. Stochastic gradient descent with Nesterov Momentum of 0.99, a weight decay of 3 × 10^−5^, and an initial learning rate of 1 × 10^−2^ were used for learning the network weights. The training time of the model was 55 h with cuDNN acceleration. The nnU-Net-3D model was trained for 625 epochs with a batch size of 2. Stochastic gradient descent with Nesterov Momentum of 0.99, a weight decay of 3 × 10^−5^, and an initial learning rate of 1 × 10^−2^ were used for learning the network weights. The training time of the model was 88 h with cuDNN acceleration.

The DL-based segmentations were post-processed to only keep the largest 3D connected component, using a pixel connectivity of 26.

### 2.3. Feature Extraction

Shape features were extracted from the 3D segmentation masks (Manual or DL-based) of PZ, non-PZ, and WP using Pyradiomics [30] (version 3.0; an open-source Python package). The following 14 shape features were extracted: Elongation, Flatness, Least Axis Length, Major Axis Length, Maximum 2D diameter (Column), Maximum 2D diameter (Row), Maximum 2D diameter (Slice), Maximum 3D diameter, Mesh Volume, Minor Axis Length, Sphericity, Surface Area, Surface Area to Volume ratio, and Voxel Volume. A detailed description of the features can be found at [31].

### 2.4. Investigation of Reproducibility

Reproducibility is defined as the “variability in measurements made on the same subject, but under changing conditions” [32]. The variability and reproducibility are inversely related, i.e., the higher the variability, the lower the reproducibility. In this work, scan 1 and scan 2 were performed on the same patients, but at different time points and using different scanning procedures.

To investigate the reproducibility, all extracted features from the two scans of 62 patients’ scans, using the manual and post-processed DL-based segmentations, were included. The reproducibility for each of the 14 shape features was investigated, separately, for each of the CNNs and compared to that of the corresponding feature from the manual segmentations. Furthermore, the DL-based segmentation performance and segmentation volume (Voxel Volume feature) in scan 1 and scan 2 were compared to those of manual segmentations.

In addition, the reproducibility results were compared to the corresponding results where (1) the post-processing step was excluded, and (2) patients with a poor segmentation quality score were excluded. To enable the last comparison, our previously proposed automated segmentation quality control system (SQCS) [33] was implemented, and the patients with a quality score of less than 85 for scan 1 or/and scan 2 were excluded from further analysis. As per [33], the SQCS was implemented using pre-processed T2W images and WP segmentations.

### 2.5. Statistical Analysis

The dice similarity coefficient (DSC) [34], between manual and DL-based segmentations, was calculated as a metric of segmentation performance.

The two-way random, single score intra-class correlation coefficient (ICC) [35,36] was used to measure the inter-scan reproducibility of each feature for each CNN and the manual segmentations. Statistical significance between features, from manual segmentation and each CNN, and between features, from including and excluding the post-processing step, was based on overlapping 95% confidence intervals (CI) [37].

The paired Wilcoxon signed rank test [38], followed by Benjamini–Hochberg correction for multiple testing [39], was used to assess the differences in DSC, the ICC values between VOIs, and segmentation volume between networks and scans.

The Bland–Altman analysis [40] and Spearman’s rank test [38] were performed to assess the correlation between the segmentation volumes for scan 1 and scan 2 and between the segmentation volumes of each of the CNNs and the manual segmentations in scan 1 and scan 2.

To assess the difference in feature reproducibility, before and after implementing the SQCS, a permutation test [38] with 1000 runs was performed for each CNN. In each of these 1000 runs, the ICC value was calculated after randomly excluding the same number of cases as excluded by the SQCS. The improvement in ICC, after applying the SQCS, was considered significant if less than 50/1000 randomly permuted values were higher or equal to the ICC after the SQCS implementation.

MATLAB R2019b (MathWorks, Natick, MA, USA) was used for statistical analysis.

## 3. Results

**Figure 1:**
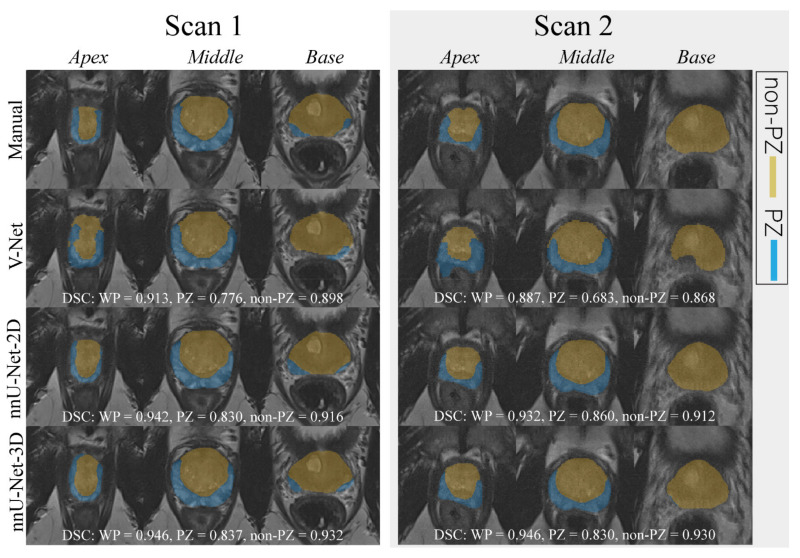
The middle slice for the whole prostate, apex, and base of a randomly selected case was segmented (peripheral zone (PZ) and the remaining prostate zones (non-PZ)) by different approaches for scan 1 and 2. For each network, the dice similarity coefficient (DSC) of the 3D segmented volume is reported for the whole prostate gland (WP), PZ, and non-PZ.

An example case segmented with the three investigated CNNs is shown in Figure 1.

**Figure 2:**
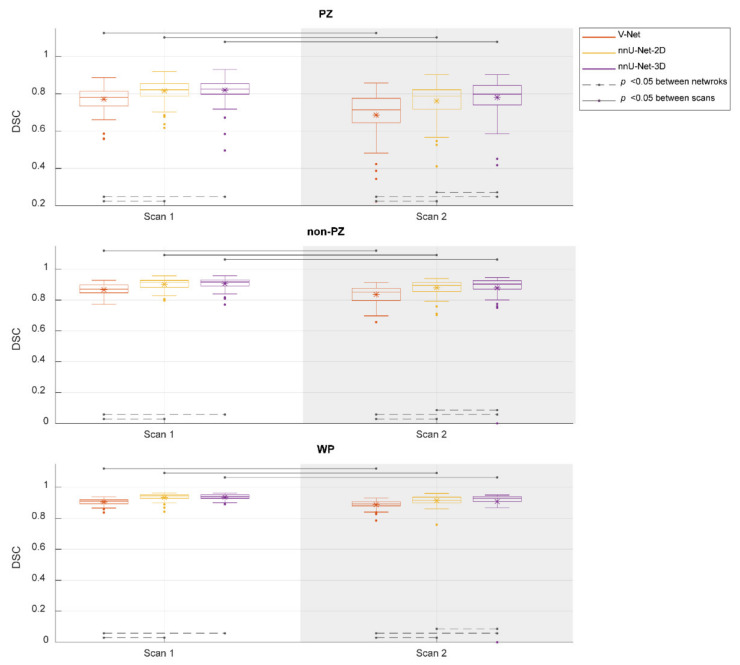
The performance (dice similarity coefficient-DSC) of the segmentation methods for the whole prostate gland (WP), peripheral zone (PZ), and the remaining prostate zones (non-PZ). The Manual segmentations were considered as reference. The means are denoted by 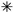, while the outliers are denoted by ●.

Figure 2 shows the performance of the investigated CNNs segmentations. The median DSCs were 0.781, 0.821, and 0.825 in PZ; 0.871, 0.916, and 0.917 in non-PZ; 0.909, 0.937, and 0.940 in WP for V-Net, nnU-Net-2D, and nnU-Net-3D, respectively, in scan 1; 0.714, 0.788, and 0.798 in PZ; 0.853, 0.896 and 0.904 in non-PZ; 0.893, 0.917, and 0.929 in WP for V-Net, nnU-Net-2D, and nnU-Net-3D, respectively, in scan 2. Median of DSC difference between the scans (scan 2–scan 1) was −9.49%, −4.06% and −3.65% in PZ; −3.12%, −1.80% and −1.08% in non-PZ; −1.98%, −1.95% and −1.39% in WP for V-Net, nnU-Net-2D and nnU-Net-3D, respectively. V-Net performed significantly lower (*p* < 0.001) than nnU-Net-2D and nnU-Net-3D in both of the scans and all of VOIs. nnU-Net-3D performed significantly higher (*p* < 0.01) than nnU-Net-2D in scan 2 for all of VOIs. In addition, each of the CNNs performed significantly lower (*p* < 0.001) in scan 2 compared to scan 1.

**Figure 3:**
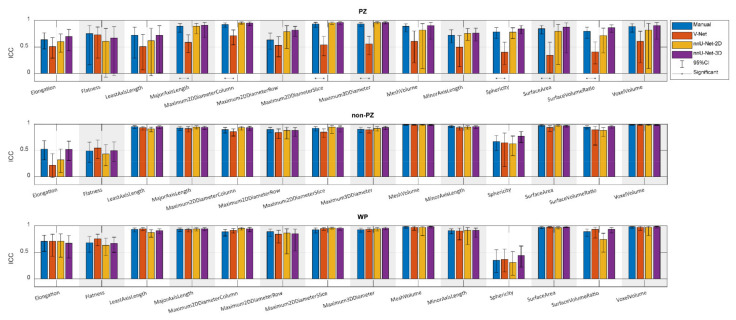
The single score intra-class correlation coefficient (ICC), with the 95% confidence interval (95% CI), of the shape features extracted from the whole prostate gland (WP), peripheral zone (PZ), and the remaining prostate zones (non-PZ) for the investigated methods, where the segmentation post-processing step was included, and the segmentation quality control system was not implemented.

Figure 3 shows the ICCs from the extracted shape features from scan 1 and scan 2, where the segmentation post-processing step was included, and the segmentation quality control system was not implemented, demonstrating that the reproducibility of DL-based segmentation is comparable to manual segmentation for all networks (14/14 features), except for V-Net in PZ (7/14 features). In both manual and DL-based segmentations, Elongation, Flatness, and Sphericity had a remarkably lower ICC than the other features in WP and non-PZ. nnU-Net-3D showed higher reproducibility than the rest of the CNNs with a median difference in ICC equal to 54.03% and 9.06% in PZ; 3.95% and 0.38% in non-PZ; 0.95% and 1.09% in WP with V-Net and nnU-Net-2D, respectively. Additionally, in most cases, feature reproducibility in the non-PZ and WP was higher than in the PZ. V-Net had significantly higher (*p* < 0.01) ICCs in non-PZ and WP compared to PZ.

**Figure A1:**
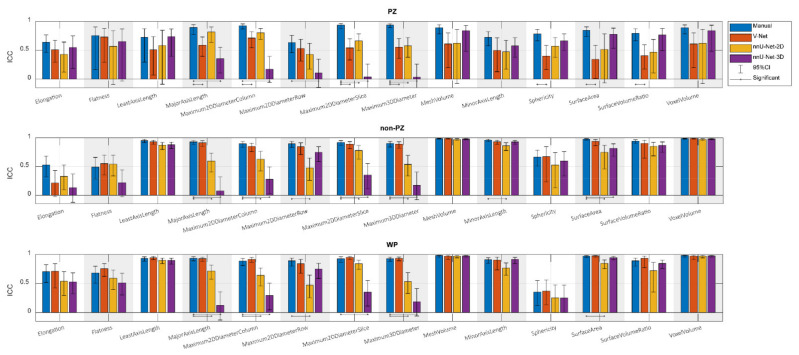
The single score intra-class correlation coefficient (ICC) with its 95% confidence interval (95%CI) of the shape features extracted from the whole prostate gland (WP), peripheral zone (PZ) and the remaining prostate zones (non-PZ) for the investigated methods, where the segmentation post-processing step was skipped and the segmentation quality control system was not implemented.

Comparing reproducibility when including (Figure 3) and excluding (Figure A1) the segmentation post-processing step, while SQCS was not implemented in any of them, shows that the reproducibility is remarkably enhanced when including the segmentation post-processing step. The ICC, after including the segmentation post-processing step, was significantly better in (4/14) features for V-Net in non-PZ; (14/14), (12/14), and (13/14) features for nnU-Net-2D in PZ, non-PZ, and WP, respectively; (13/14), (14/14), and (13/14) features for nnU-Net-3D in PZ, non-PZ, and WP, respectively.

**Figure A2:**
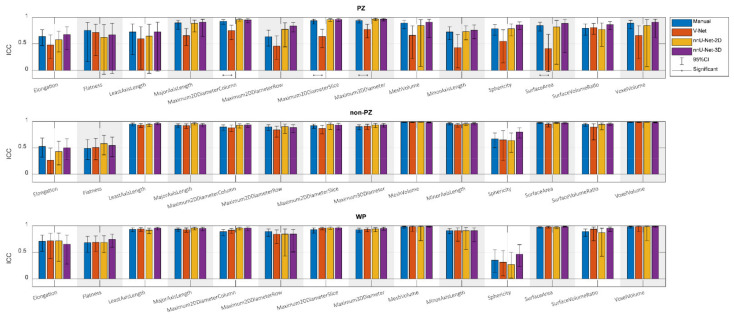
The single score intra-class correlation coefficient (ICC) with its 95% confidence interval (95%CI) of the shape features extracted from the whole prostate gland (WP), peripheral zone (PZ) and the remaining prostate zones (non-PZ) for the investigated methods, where the segmentation post-processing step was included and the segmentation quality control system was implemented. The patients with a quality score less than 85 for scan 1 or/and scan 2 were excluded.

Similarly, the reproducibility was increased with the SQCS implementation (Figure A2) compared to no implementation (Figure 3); the segmentation post-processing step was included in both cases. After implementing the SQCS, 10, 11, and 6 patient’s segmentations were excluded from V-Net, nnU-Net-2D, and nnU-Net-3D, respectively. The ICC after implementing the SQCS was significantly better in (3/14), (2/14), and (3/14) features for V-Net in PZ, non-PZ, and WP, respectively; in (7/14) and (2/14), features for nnU-Net-2D in non-PZ, and WP, respectively; in (1/14) and (5/14) features for nnU-Net-3D in non-PZ and WP, respectively.

**Figure 4:**
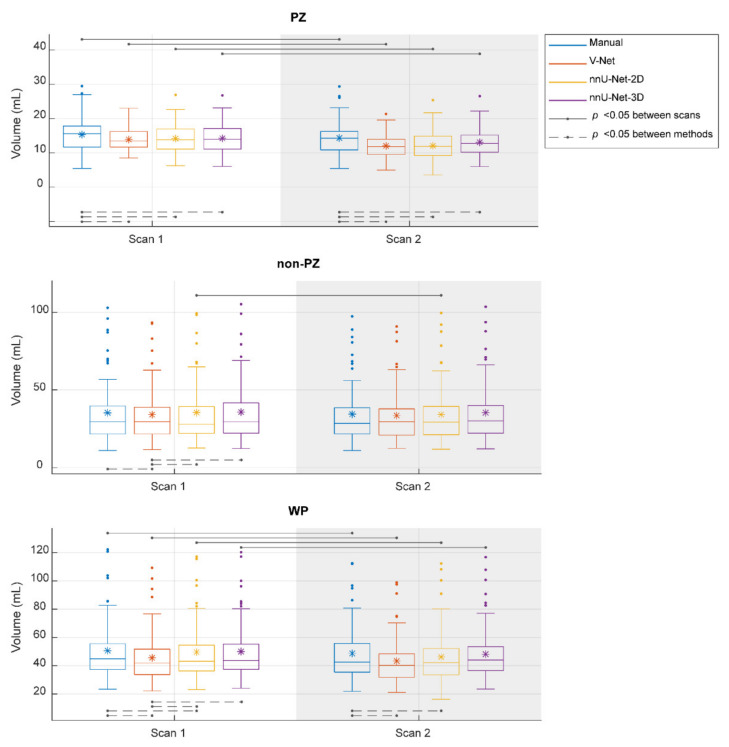
The segmented volume of the whole prostate gland (WP), peripheral zone (PZ), and the remaining prostate zones (non-PZ) from the investigated methods in scan 1 and scan 2. The means are denoted by 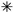, while the outliers are denoted by ●.

**Figure A3:**
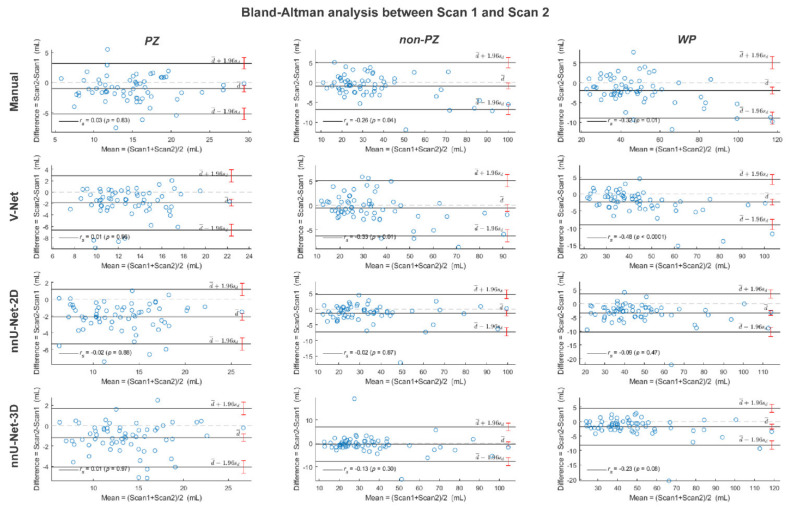
The Bland-Altman plots for the agreement between scan 1 and scan 2 volumes from the whole prostate gland (WP), peripheral zone (PZ) and the remaining prostate zones (non-PZ) for the investigated methods. The 95% confidence interval limits for mean and agreement limits are denoted by Ι, while the cases are denoted by ○.

**Figure A4:**
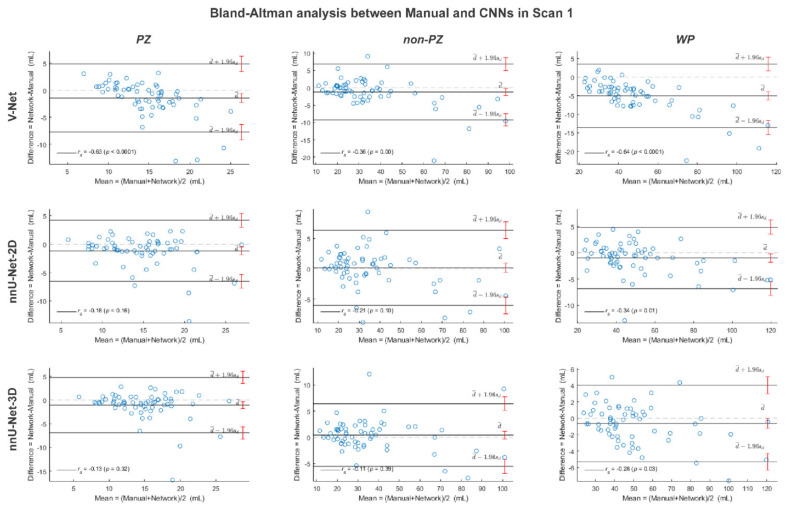
The Bland-Altman plots for the agreement between manual and rest of the investigated methods volumes from the whole prostate gland (WP), peripheral zone (PZ) and the remaining prostate zones (non-PZ) in scan 1. The 95% confidence interval limits for mean and agreement limits are denoted by Ι, while the cases are denoted by ○.

**Figure A5:**
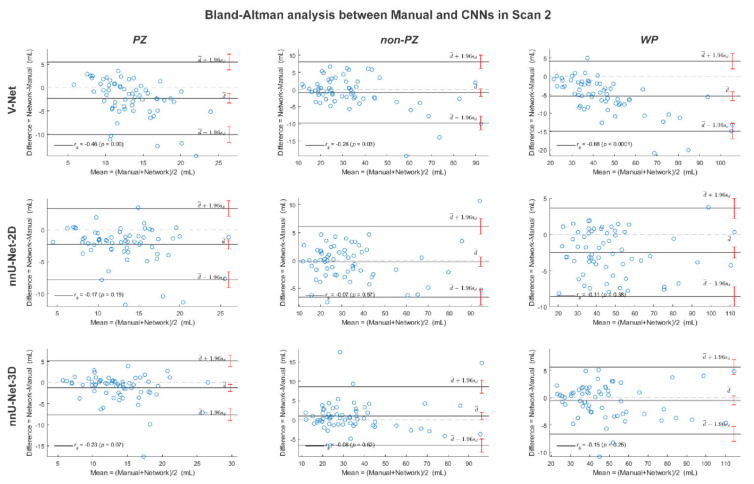
The Bland-Altman plots for the agreement between manual and rest of the investigated methods volumes from the whole prostate gland (WP), peripheral zone (PZ) and the remaining prostate zones (non-PZ) in scan 2. The 95% confidence interval limits for mean and agreement limits are denoted by Ι, while the cases are denoted by ○.

The segmented volume (Voxel Volume feature) was further investigated, as it is an important and in-use biomarker for multiple clinical applications [41,42,43]. Its ICC score, when the segmentation post-processing step was included and the SQCS was not implemented, was 0.888, 0.607, 0.819, and 0.903 in PZ; 0.988, 0.967, 0.986, and 0.983 in non-PZ; 0.982, 0.975, 0.973, and 0.984 in WP for manual, V-Net, nnU-Net-2D, and nnU-Net-3D, respectively. Figure 4 shows that the segmented volume was significantly lower in scan 2, compared to scan 1, for all the methods in PZ and WP (*p* < 0.001) and for nnU-Net-2D in non-PZ (*p* = 0.003). Bland–Altman analysis shows a similar bias for manual and DL-based methods (Figure A3). Median of volume difference between the scans (scan 2–scan 1) was −4.33%, −3.58%, −5.80%, and −3.32% in WP for manual, V-Net, nnU-Net-2D, and nnU-Net-3D, respectively. It also shows a small bias between the volumes of the DL-based and manual segmentations in scan 1 (Figure A4) and scan 2 (Figure A5). It was noticed that PZ has higher bias between scans and methods than non-PZ and WP. V-Net has also showed a slightly higher bias between scans and methods than nnU-Net-2D and nnU-Net-3D.

## 4. Discussion

VOI segmentation is an essential step in CAD systems. DL-based methods provide good performance for prostate segmentation, but little is known about their reproducibility. The reproducibility of radiomics shape features can be used as an indicator of the segmentation reproducibility. Therefore, in this paper, we investigated the reproducibility of the shape features extracted from DL-based segmentations of the WP, PZ, and non-PZ on T2W MR images, acquired with short time intervals (median = 7 days), and compared them to those of manual segmentations. Prostate gland volume is proportionally related to benign enlargement [44] and inversely related to prostate cancer [45]. Both of those conditions usually require long time to develop, thus no significant change in prostate gland volume is expected during a short time interval. Shape features, such as prostate volume, used to measure the PSA-density (PSA level/prostate volume) [46], are already part of today’s clinical risk calculators for prostate cancer [8] and will likely play an important role in future radiomics-based clinical applications. For clinical applications based on multiple scans in time, like active surveillance, it is key that extracted features are both accurate and reproducible.

The DSC values were in line with those expected from the literature [16,18], indicating that the trained networks have state-of-the-art performance. nnU-Net-3D had the best overall segmentation accuracy, while V-Net showed the lowest segmentation accuracy comparable to the manual segmentations. This work extends previous studies, showing the excellent performance of nnU-Net, specifically the 3D volume basis model, on a wide variety of medical image segmentation tasks [16]. The DSC values were slightly lower in scan 2 compared to scan 1. This is probably due to the nature of the segmentation training set, which consisted of cases acquired with a scan protocol similar to that of scan 1.

Based on ICCs of the shape features, nnU-Net-2D and nnU-Net-3D were shown to have comparable reproducibility to manual segmentations in all VOIs. WP and non-PZ showed higher ICCs compared to PZ, which was expected due to the low PZ segmentation performance. nnU-Net-3D provided higher ICCs compared to the other CNNs, which was expected as it had the highest segmentation performance among CNNs. Overall, the results show that DL-based segmentation methods can generate highly intra-patient reproducible masks for T2W images of the prostate. Good reproducibility gives potential for picking up changes in the prostate when they appear, an important step towards the clinical implementation of prostate CAD systems, based on multiple T2W MRI scans.

Including a post-processing step to the segmentation, where only the largest connected component in 3D volume is kept, was shown to remarkably enhance the features reproducibility. Similarly, the implementation of the SQCS significantly increased the reproducibility. Therefore, the implementation of these two post-processing steps in a CAD system pipeline is recommended to assure highly reproducible shape features. In clinical applications, the cases with low segmentation quality score, predicted by the SQCS, should be either referred to a radiologist for manual intervention or re-segmented using another CNN.

One possible explanation for the lower ICC of Elongation, Flatness, and Sphericity in WP and non-PZ is that the prostate gland in scan 2 was potentially compressed due to a guiding probe for the biopsy needle inside the patient’s rectum during the image acquisition. Moreover, the patients were scanned in prone position during scan 2, in contrast to scan 1, where they were scanned in supine position. The probe and the prone position would, indeed, not alter the volume of the prostate but might deform its shape slightly. In their study, Osman et al. [47] have investigated the endorectal coil effect on the WP volume and shape during prostate T2W MRI and concluded that, despite shape deformation, there is no significant change in the WP volume between including and excluding the endorectal coil. Although the needle guiding probe differs from the endorectal coil, its impact may be expected to be similar. In addition, the prostate gland might deform between scans due to other factors, e.g., different bladder/bowel loading, which were not taken into account in this study. The shape deformation may have had an impact on the decision of including or excluding a slice from the segmentation. We noticed that, overall, scan 2 had a lower number of segmented slices than scan 1. Median of the segmented slices number was 14, 14, 14, and 14 in WP for manual, V-Net, nnU-Net-2D, and nnU-Net-3D, respectively, in scan 1 and 13, 13.5, 13, and 14 in WP for manual, V-Net, nnU-Net-2D, and nnU-Net-3D, respectively, in scan 2. Although the difference between the numbers is small (≈1 slice), it will influence the segmented volumes, which were indeed slightly lower in scan 2 than in scan 1.

The reproducibility of the segmented volume might be the most important among the 14 investigated features. WP volume is used by the radiologist to measure the PSA-density, which is part of today’s clinical risk calculators [8], and can be used as a biomarker to evaluate prostate cancer progression and the need for re-biopsy [43]. An alternative biomarker to the traditional PSA-density is the zonal adjusted PSA-density, which depends on the segmented volume from various prostate gland zones, i.e., non-PZ volume [48,49]. Our study shows that the segmented volume feature is highly reproducible, and in agreement with manual volumes on both zonal and whole prostate-level.

In their work, Schwier et al. [22] used manual segmentations to assess the reproducibility of radiomics features on prostate T2W MR images. Their focus was mainly on the reproducibility of the radiomics textural features under different settings, but they have also included results on some of the shape features reproducibility. Although there is some similarity between their work and ours, our work focused on the reproducibility of DL-based segmentations. Like in our work, Schwier et al. showed that the reproducibility of shape features is high. Furthermore, they showed that the segmented volume reproducibility is higher in WP than in PZ, which was also in line with our findings. The high ICC values found in this work suggest that all the shape features, except for Elongation, Flatness, and Sphericity, extracted using DL-based segmentation methods, can be used in clinical applications based on multiple scans without being concerned about their reproducibility.

In this work, we used a dataset from prostate cancer patients referred and scanned according to prevailing guidelines. Consequently, the results represent the reproducibility of the DL-based segmentations in a real clinical setting. Nevertheless, our study has some limitations. The patient cohort was relatively small, and it was obtained from a single center. Conducting a multicenter study in the future might give additional insight on the reproducibility of DL-based segmentation across institutions. Moreover, the manual segmentations in this study have been performed by one reader. A set of manual segmentations, where multiple readers are included, will facilitate additional comparisons, which might provide us with more information, but this can be considered for a future work.

## 5. Conclusions

We investigated the reproducibility of the shape features, extracted from DL-based segmentations, of the prostate gland and zones on T2W MR images acquired with short time intervals. The reproducibility of the best-performing DL-based prostate segmentation methods is comparable to that of manual segmentations, which is important for clinical applications, based on multiple scans in time.

## Figures and Tables

**Table 1:** Summary of MRI scanning parameters.

	Investigation Set		Training Set
	Scan 1	Scan 2	
Repetition time (ms)	4800–8921	5660–7740	4450–9520
Echo time (ms)	101–104	101–104	101–108
Flip angle (degree)	152–160	152–160	145–160
Number of averages	3	3–6	1–3
Matrix size	320 × 320–384 × 384	320 × 320–384 × 384	320 × 320–384 × 384
Slices	24–30	17–24	24–34
Slice thickness (mm)	3	3	3–3.5
In plane resolution (mm^2^)	0.5 × 0.5–0.6 × 0.6	0.5 × 0.5–0.6 × 0.6	0.5 × 0.5–0.6 × 0.6

## Data Availability

The dataset used in this study are not publicly available due to specific institutional requirements governing privacy protection.
